# Patterns of coronal and sagittal deformities in adolescent idiopathic scoliosis

**DOI:** 10.1186/s12891-020-03937-4

**Published:** 2021-01-08

**Authors:** Trixie Mak, Prudence Wing Hang Cheung, Teng Zhang, Jason Pui Yin Cheung

**Affiliations:** 1 Department of Orthopaedics and Traumatology, The University of Hong Kong, Pokfulam Hong Kong SAR, China; 2 Department of Orthopaedics and Traumatology, The University of Hong Kong Shenzhen Hospital, Shenzhen, China

**Keywords:** Adolescent idiopathic scoliosis, Pelvic incidence, Pelvic tilt, Sacral slope, Lumbar lordosis, Thoracic kyphosis

## Abstract

**Background:**

Thoracic scoliosis has been shown to be associated with hypokyphosis in adolescent idiopathic scoliosis (AIS). However, the relationship of sagittal spino-pelvic parameters with different coronal curve patterns and their influence on patient-perceived quality of life is unknown. This study aims to determine the association between coronal and sagittal malalignment in patients with AIS and to determine their effects on SRS-22r scores.

**Methods:**

A cross-sectional study was conducted on 1054 consecutive patients with AIS. The coronal Cobb angle, thoracic kyphosis (TK), lumbar lordosis (LL), pelvic incidence (PI), PI-LL mismatch (PI-LL), pelvic tilt (PT), and sacral slope (SS) were measured on standing radiographs. The coronal Cobb angle (mild: 10–20°; moderate: > 20–40°; severe: > 40°) and PI (low: < 35°; average: 35–50°; high: > 50°) were divided into 3 sub-groups for comparison. Relationship between coronal curve magnitudes and sagittal parameters was studied as was their association with SRS-22r scores.

**Results:**

Low PI had smaller SS (30.1 ± 8.3° vs 44.8 ± 7.7°; *p* < 0.001), PT (− 0.3 ± 8.1° vs 14.4 ± 7.5°; *p* < 0.001), and LL (42.0 ± 13.2° vs 55.1 ± 10.6°; *p* < 0.001), negative PI-LL mismatch (− 12.1 ± 13.1° vs 4.1 ± 10.5°; *p* < 0.001) as compared to large PI. There were no significant relationships with PI and TK (*p* = 0.905) or curve magnitude (*p* = 0.431). No differences in sagittal parameters were observed for mild, moderate or severe coronal Cobb angles. SRS-22r scores only correlated with coronal Cobb angle and larger Cobb angles were negatively correlated with the function, appearance and pain domains.

**Conclusions:**

The sagittal profile for AIS is associated with the pelvic parameters especially PI but not with the coronal curve pattern. All patients have a similar TK regardless of coronal curve type. However, it appears that the coronal deformity is a greater influence on quality of life outcomes especially those > 40°.

## Background

Adolescent idiopathic scoliosis (AIS) is a three-dimensional spinal deformity, consisting of lateral deviation of the vertebral column with rotation of the vertebrae, and sagittal spinal curvature disruption [1]. It is the most prevalent spine problem in adolescent patients and treatment options include observation, brace prescription, posture training, reassurance and surgery [2]. Among teenagers aged between 10 to 16, it is found that 2–4% will develop some degree of scoliosis [1].

As AIS is a three-dimensional deformity, management should not be focused only on the coronal plane. The coronal and sagittal plane deformities are coupled and thus, variations in the coronal plane may translate into sagittal plane changes [3–5]. Mac-Thiong et al [6] evaluated the sagittal alignment of 160 patients with AIS and found less thoracic kyphosis in thoracic major curves compared to lumbar curves, and lumbar curve patients tend to have larger lumbar lordosis. Limited by a small sample size, no differences were found between sacral slope, pelvic incidence and pelvic tilt among the groups, so this study suggested no specific sagittal patterns for different types of coronal plane deformities. Several other small-scale studies also investigated how different sagittal parameters interact in scoliotic patients, though no conclusion was drawn regarding the relationship between the coronal and sagittal plane deformities [7–10].

Individualized evaluation on sagittal alignment is needed to better understand the disease as it may influence patient quality of life [11] and perhaps the likelihood for developing back pain [2]. The refined 22-item Scoliosis Research Society questionnaire (SRS-22r) is a well-established tool for assessment of quality of life in patients with AIS [11, 12]. However, its utility in assessment of different coronal and sagittal parameters in the AIS population is not well understood with particular magnitudes of coronal curve severity and spino-pelvic alignment. Thus, the objective of this study is to determine the relationship between the coronal deformity and sagittal spino-pelvic alignment in patients with AIS and whether different coronal and sagittal patterns affect quality of life outcome measures.

## Methods

### Study design

In this cross-sectional study, posteroanterior (PA) and lateral radiographs were collected from 1251 consecutive patients with AIS who visited a tertiary referral scoliosis clinic from October 2018 to February 2019. All patients included in the study were under observation without active treatment. Only adolescent patients (10–18 years old) were included in the study. Patients who were not diagnosed as AIS, underwent surgery and were not in the age range of 10–18 were excluded. Ethics was approved by the local institutional review board (UW 15–596). All patients had written informed consent regarding their data used for study.

### Study parameters

All radiographs were obtained with patients standing and out of brace if applicable. All measurements were made with the ImageJ software (64-bit Java 1.8.0) (National Institutes of Health, Maryland, USA) [13]. Measurements were all made by two investigators independently and blinded to the clinical information to avoid bias. The list of patients for measurement were randomly allocated and provided by another investigator. An average score was used for any measurement with < 5 degrees of difference. Any difference beyond 5 degrees was discussed between the investigators with a final consensus on the measurement used for analysis. The cut-off of 5 degrees was used based on documented radiographic measurement errors in a scoliotic curve [14].

The coronal Cobb angles of the major and minor curve(s) were measured on PA radiographs. Based on the curve pattern, patients were separated into groups according to the location of major curve and the number of structural curves. Structural curves were considered for curves with a clinical hump on forward bending test and evidence of rotation on radiographs. Groups for location of major curve included thoracic region (apex between T1 and T12) and thoracolumbar/lumbar region (apex between T12 and L4), while groups for number of structural curves included single structural curve and multiple structural curves.

On the lateral radiographs, sagittal spino-pelvic parameters were measured including lumbar lordosis (LL) and thoracic kyphosis (TK), pelvic incidence (PI), pelvic tilt (PT), and sacral slope (SS). PI is the angle between a perpendicular line from the midpoint of the sacral endplate and a line from the midpoint of the sacral endplate to the centre of femoral head in the sagittal plane [7]. PT is the angle between a line from the midpoint of the sacral endplate to the centre of femoral head and a vertical reference line from the centre of femoral head in the sagittal plane [15]. SS is the angle between a line along the sacral endplate and a horizontal reference line [16]. LL is the angle between the upper endplate of L1 and the upper endplate of S1 in sagittal plane [17]. TK represented the maximum kyphotic angle measured in the thoracic spine [18]. PI-LL mismatch was calculated as it pertains to the mismatch in spino-pelvic alignment [8, 19].

The refined 22-item Scoliosis Research Society (SRS-22r) patient questionnaire was used to evaluate patients’ function, pain, appearance, mental health and satisfaction on treatment. The total score is 5, with higher scores representing higher quality of life [20]. Its minimum clinically important difference (MCID), based on a 5-point scale, has been quoted as 0.08 for function, 0.2 for pain, and 0.98 for appearance domains [21]. Mental health has no quoted minimum clinically important difference for the AIS population. Satisfaction with treatment is described and based on improvement or deterioration in domain scores. These scores were obtained immediately prior to seeing the clinician at the consultation room.

### Statistical analysis

The data was analyzed using Excel (Microsoft, Washington, USA). Shapiro-Wilk test [22] found that the data was not normally distributed. Hence, Mann-Whitney U test [23] was used to compare the sagittal values with the location of major curve and the number of structural curves. The PI and coronal Cobb angle were subclassified into three separate subgroups for further analyses with other radiological parameters using analysis of variance (ANOVA) and post-hoc pairwise comparison with significance adjusted by Bonferroni correction. Based on previous descriptions, the PI was divided into low PI (< 35°), average PI (35–50°) and high PI (> 50°) [24–26]. For coronal Cobb angle, three groups of 10–20°, > 20–40° and > 40° were used to differentiate between mild, moderate and severe curves respectively. SRS-22r scores of different groups were also compared using the Mann-Whitney U test, while the correlation between the scores and parameters were analysed using Spearman’s rank correlation coefficient. The Spearman’s rank correlation coefficient (r) was used to analyse the correlation between different sagittal parameters [27]. The correlation coefficient value was evaluated as follows: < 0.1: slight; 0.1–0.29: weak; 0.3–0.49: medium; 0.5–0.79: strong; 0.8 or above: very strong [28]. Significance level was set at *p* < 0.05.

## Results

**Fig. 1:**
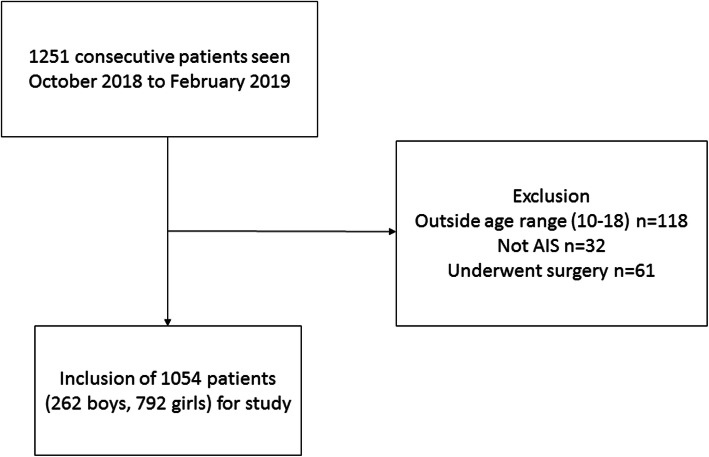
Flowchart of included patients for analysis

Of the 1251 consecutive patients seen during the study period, 118 were excluded due to the age range not within 10–18 years, 32 who were not AIS, and 61 who had underwent surgery. A total of 1054 patients (262 boys, 792 girls) were included in the study (Fig. 1) after exclusion. Of these, 602 had thoracic major curves, and 452 had thoracolumbar/lumbar major curves. There were 855 single structural curves and 199 multiple structural curves. The mean age of the study population was 14.2 ± 1.9 years and the mean coronal Cobb angle of the major curve was 27.0 ± 10.4° (range 10.1°-85.8°). Table 1 shows a comparison of coronal and sagittal parameters according to different curve locations and number of structural curves. Regarding the location of major curve, thoracic major curves had less PI-LL mismatch, smaller LL and less TK when compared to thoracolumbar/lumbar major curves but the differences remained within measurement error. Multiple structural curves tended to have larger Cobb angles than single structural curves. Multiple structural curves group also showed larger PI, larger SS, less PI-LL mismatch, and less TK than the single structural curve group, but again these differences remained within measurement error.

**Table 1:** Radiographic parameters according to location and number of structural curves

	Location of major curve			Number of structural curves			Overall
	Thoracic mean ± SD (range) (°)	Thoraco-lumbar/ Lumbar mean ± SD (range) (°)	***P***-value	Single mean ± SD (range) (°)	Multiple mean ± SD (range) (°)	*P*-value	mean ± SD (range) (°)
**PI**	47.7 ± 12.1 (3.6–97.2)	47.3 ± 11.4 (15.8–85.9)	0.390	47.2 ± 11.8 (3.6–97.2)	49.1 ± 11.7 (19.4–87.1)	0.022*	47.5 ± 11.8 (3.6–97.2)
**PT**	9.2 ± 8.9 (− 30.8–44.4)	8.7 ± 8.2 (− 17.6–33.9)	0.210	8.8 ± 8.8 (− 30.8–44.4)	9.4 ± 8.1 (− 13.6–29.7)	0.260	9.0 ± 8.6 (− 30.8–44.4)
**SS**	38.6 ± 9.3 (12.6–69.3)	38.6 ± 8.8 (9.4–65.6)	0.280	38.3 ± 9.2 (9.4–69.31)	39.6 ± 8.4 (21.5–65.6)	0.041*	38.6 ± 9.1 (9.4–69.3)
**PI-LL**	−0.96 ± 12.7 (− 53.2–36.3)	− 3.2 ± 11.6 (− 40.0–29.9)	0.002*	−2.3 ± 12.5 (− 53.2–36.3)	−0.29 ± 11.2 (− 28.9–34.7)	0.049*	−1.9 ± 12.3 (− 53.2–36.3)
**LL**	48.7 ± 11.9 (14.2–90.0)	50.5 ± 12.2 (9.1–83.5)	0.003*	49.5 ± 12.4 (9.1–90.0)	49.4 ± 10.7 (18.7–78.2)	0.497	49.5 ± 12.1 (9.1–90)
**TK**	17.3 ± 11.0 (− 23.8–71.1)	19.4 ± 9.7 (− 24.7–51.0)	< 0.001*	18.6 ± 10.3 (− 10.6–71.1)	16.7 ± 11.5 (− 24.7–58.9)	0.012*	18.2 ± 10.5 (− 24.7–71.1)
**Major curve**	27.2 ± 10.7 (10.1–85.8)	26.2 ± 9.3 (10.4–69.8)	0.091	24.2 ± 8.2 (10.1–70.7)	39.1 ± 10.2 (24.6–85.8)	< 0.001*	27.0 ± 10.4 (10.1–85.8)
**Minor curve 1**	21.4 ± 9.7 (3.6–79.9)	21.5 ± 8.4 (0.8–61.1)	0.190		33.1 ± 7.8 (25.0–79.9)		21.4 ± 9.2 (0.8–79.9)
**Minor curve 2**	17.7 ± 6.8 (1.8–31.9)	19.5 ± 8.1 (7.7–33.3)	0.360		29.1 ± 2.5 (25.5–33.3)		18.1 ± 7.0 (1.8–33.3)

PI: pelvic incidence; PT: pelvic tilt; SS: sacral slope; LL: lumbar lordosis; TK: thoracic kyphosis; SD: standard deviation

*Significant correlation (*p* < 0.05)

Curve magnitude did not appear to influence sagittal alignment parameters (Table 2). TK was also similar in different sub-groups but were all hypokyphotic. There was close matching of LL with PI within the three groups. With stratification of PI (Table 3), most patients with AIS did not present with high PI. All three groups had different sagittal spino-pelvic patterns. Those with a low PI had more vertical sacrum and smaller SS (30.1 ± 8.3° vs 36.1 ± 7.0° and 44.8 ± 7.7°; *p* < 0.001) and PT (− 0.3 ± 8.1° vs 7.2 ± 6.5° and 14.4 ± 7.5°; *p* < 0.001), reduced LL (42.0 ± 13.2° vs 47.1 ± 10.9° and 55.1 ± 10.6°; *p* < 0.001), negative PI-LL mismatch (− 12.1 ± 13.1° vs − 3.9 ± 10.9° and 4.1 ± 10.5°; *p* < 0.001) than average and high PI. The TK was similar between the three groups (*p* = 0.905). The PI was independent of coronal curvature (*p* = 0.431).

**Table 2:** Relationship between radiographic parameters and curve magnitude

	Major curve 10–20° mean (°) ± SD (range) ***n*** = 282	Major curve > 20–40° mean (°) ± SD (range) ***n*** = 668	Major curve > 40° mean (°) ± SD (range) ***n*** = 104	*p*-value^	Post-hoc pairwise comparison
	**Group 1**	**Group 2**	**Group 3**		
**PI**	46.7 ± 10.5 (22.1–76.2)	47.6 ± 12.0 (15.8–97.2)	49.7 ± 13.0 (3.6–87.1)	0.076	
**PT**	8.7 ± 8.2 (− 20.5–35.3)	9.0 ± 8.7 (− 17.6–44.4)	9.4 ± 9.5 (− 30.8–28.1)	0.744	
**SS**	37.9 ± 8.4 (13.4–62.6)	38.6 ± 9.2 (11.4–69.3)	40.4 ± 9.5 (9.4–64.6)	0.070	
**PI-LL**	−2.9 ± 11.5 (− 46.2–26.9)	−1.7 ± 12.3 (− 44.3–36.3)	−0.1 ± 14.1 (− 53.2–29.9)	0.086	
**LL**	49.6 ± 11.8 (17.0–81.2)	49.3 ± 12.0 (13.7–90)	49.9 ± 13.2 (9.1–89.8)	0.913	
**TK**	19.4 ± 10.1 (− 4.1–71.1)	17.8 ± 10.4 (− 24.7–69.3)	17.3 ± 12.3 (− 23.8–58.9)	0.029*	Group 3 vs 2: 1.000 Group 3 vs 1: 0.080 Group 2 vs 3: 0.065
**Major curve**	16.5 ± 2.5 (10.1–20.0)	27.8 ± 5.3 (20.1–40.0)	49.9 ± 8.8 (40.1–85.8)	< 0.001*	Group 3 vs 2: < 0.001* Group 3 vs 1: < 0.001* Group 2 vs 3: < 0.001*

PI: pelvic incidence; PT: pelvic tilt; SS: sacral slope; LL: lumbar lordosis; TK: thoracic kyphosis; SD: standard deviation

^Kruskal-Wallis test

*indicates statistically significant difference in mean rank thoracic kyphosis

**Table 3:** Relationship between radiographic parameters and pelvic incidence

	PI < 35 mean ± SD (range) ***n*** = 135	PI 35–50 mean ± SD (range) ***n*** = 522	PI > 50 mean ± SD (range) ***n*** = 397	*p*-value^	Post-hoc pairwise comparisonwith significance adjusted byBonferroni correction
	**Group1** **Low PI**	**Group 2** **Average PI**	**Group 3** **High PI**		
**PI**	29.8 ± 5.2 (3.6–34.9)	43.2 ± 4.2 (35.0–50.0)	59.2 ± 8.2 (50.1–97.2)	< 0.001*	Group 1 vs 2: < 0.001* Group 1 vs 3: < 0.001* Group 2 vs 3: < 0.001*
**PT**	−0.3 ± 8.1 (− 30.8–21.3)	7.2 ± 6.5 (− 10.8–26.2)	14.4 ± 7.5 (− 4.2–44.4)	< 0.001*	Group 1 vs 2: < 0.001* Group 1 vs 3: < 0.001* Group 2 vs 3: < 0.001*
**SS**	30.1 ± 8.3 (9.4–50.9)	36.1 ± 7.0 (14.6–57.0)	44.8 ± 7.7 (23.3–69.3)	< 0.001*	Group 1 vs 2: < 0.001* Group 1 vs 3: < 0.001* Group 2 vs 3: < 0.001*
**PI-LL**	− 12.1 ± 13.1 (− 53.0–16.3)	−3.9 ± 10.9 (− 53.2–29.2)	4.1 ± 10.5 (− 23.1–36.3)	< 0.001*	Group 1 vs 2: < 0.001* Group 1 vs 3: < 0.001* Group 2 vs 3: < 0.001*
**LL**	42.0 ± 13.2 (9.1–75.1)	47.1 ± 10.9 (14.2–90.0)	55.1 ± 10.6 (21.5–87.1)	< 0.001*	Group 1 vs 2: < 0.001* Group 1 vs 3: < 0.001* Group 2 vs 3: < 0.001*
**TK**	18.5 ± 10.3 (− 4.1–51.3)	18.2 ± 10.7 (− 24.7–69.3)	18.1 ± 10.3 (− 3.8–71.1)	0.905	
**Major curve**	27.0 ± 10.4 (10.2–67.6)	26.6 ± 10.5 (10.3–85.8)	27.5 ± 10.4 (10.1–69.8)	0.431	

PI: pelvic incidence; PT: pelvic tilt; SS: sacral slope; LL: lumbar lordosis; TK: thoracic kyphosis; SD: standard deviation

^ Kruskal-Wallis test

* denotes statistical significance *p* < 0.05

Age appeared to have positively weak correlation with the function (r = 0.11) and appearance (r = 0.12) domains and have slight negative correlation with the pain domain (r = − 0.095) of the SRS-22r. The mean SRS-22r scores (Table 4) were higher for the milder curves as compared to moderate and severe curves (4.44 ± 0.35 vs 4.37 ± 0.40 and 4.25 ± 0.38; *p* = 0.002). This similar difference was observed in the domains of function (4.81 ± 0.32 vs 4.76 ± 0.39 and 4.68 ± 0.39; *p* = 0.020), pain (4.75 ± 0.36 vs 4.67 ± 0.41 and 4.54 ± 0.44; p = 0.002) and appearance (3.89 ± 0.62 vs 3.85 ± 0.63 and 3.58 ± 0.65; *p* = 0.002) as well. The function and pain domains reached MCID when comparing mild and severe curves. For the relationship between SRS-22r scores and the PI, no significant differences were observed (Table 5).

**Table 4:** Mean values of SRS-22r scores based on curve magnitude

	Major curve 10–20° mean ± SD	Major curve > 20–40° mean ± SD	Major curve > 40° mean ± SD	Intergroup comparison	*p*-value^	Post-hoc pairwise comparison with significance adjusted by Bonferroni correction
	**Group 1**	**Group 2**	**Group 3**			
**Function**	4.81 ± 0.32	4.76 ± 0.39	4.68 ± 0.39		0.020*	Group 3 vs 2: 0.069 Group 3 vs 1: 0.016* Group 2 vs 1: 0.718
**Pain**	4.75 ± 0.36	4.67 ± 0.41	4.54 ± 0.44		0.002*	Group 3 vs 2: 0.028* Group 3 vs 1: 0.002* Group 2 vs 1: 0.228
**Appearance**	3.89 ± 0.62	3.85 ± 0.63	3.58 ± 0.65		0.002*	Group 3 vs 2: 0.002* Group 3 vs 1: 0.002* Group 2 vs 1: 1.000
**Mental health**	4.37 ± 0.58	4.32 ± 0.61	4.28 ± 0.61		0.542	
**Satisfaction**	3.93 ± 0.68	3.76 ± 0.79	3.74 ± 0.69		0.285	
**Total**	4.44 ± 0.35	4.37 ± 0.40	4.25 ± 0.38		0.002*	Group 3 vs 2: 0.013* Group 3 vs 1: 0.001* Group 2 vs 1: 0.388

SD: standard deviation

^ Kruskal-Wallis test

*indicates statistically significant difference in mean rank

**Table 5:** Mean values of SRS-22r scores based on pelvic incidence

	PI < 35 mean ± SD	PI 35–50 mean ± SD	PI > 50 mean ± SD	Intergroup comparison	*p*-value^
	Group 1 Low PI	Group 2 Average PI	Group 3 High PI		
**Function**	4.80 ± 0.34	4.76 ± 0.38	4.75 ± 0.38		0.811
**Pain**	4.68 ± 0.38	4.67 ± 0.41	4.67 ± 0.41		0.974
**Appearance**	3.92 ± 0.59	3.81 ± 0.61	3.83 ± 0.67		0.388
**Mental health**	4.34 ± 0.66	4.34 ± 0.60	4.30 ± 0.60		0.695
**Satisfaction**	3.89 ± 0.66	3.82 ± 0.76	3.71 ± 0.79		0.504
**Total**	4.41 ± 0.36	4.37 ± 0.38	4.36 ± 0.41		0.755

SD: standard deviation; PI: pelvic incidence

^ Kruskal-Wallis test

## Discussion

**Fig. 2:**
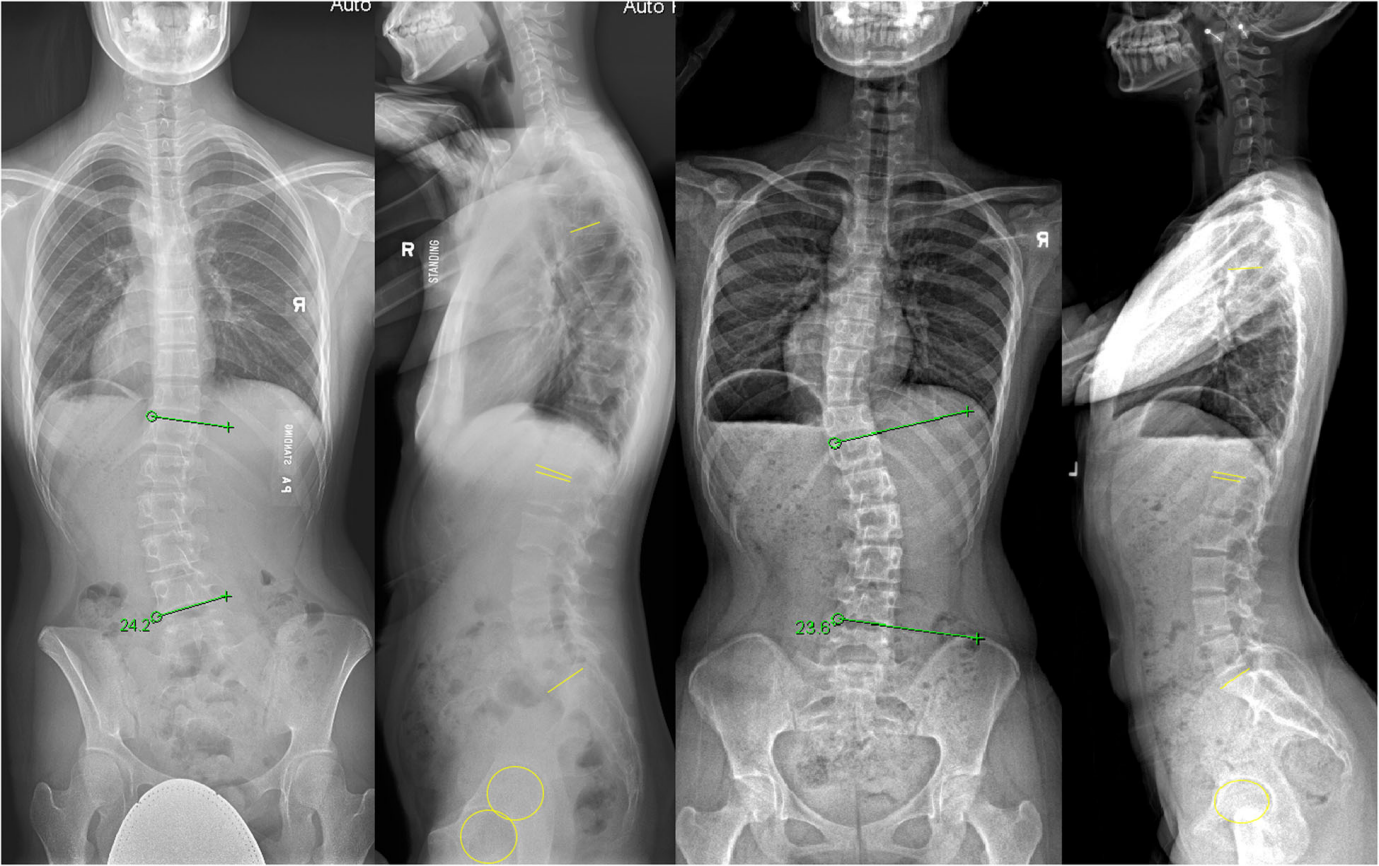
Examples of two patients with similar lumbar coronal deformity but markedly different sagittal alignment. For the first patient with (**a**) lumbar curve of 24.2° at T12-L4, (**b**) sagittal parameters included lumbar lordosis of 50.8°, reciprocal thoracic kyphosis of 39.4°, pelvic incidence of 60.2°, pelvic tilt of 25.6°, sacral slope of 34.6°, Pelvic incidence – lumbar lordosis of 9.4°. Despite a similar (**c**) coronal curve magnitude (23.6° at T12-L4), there was a (**d**) greater mismatch between pelvic and spinal parameters (− 14.2°) with hypokyphotic thoracic spine (15.4°). Other sagittal parameters included pelvic incidence of 32.9°, pelvic tilt of − 2.8°, sacral slope of 35.7°, and lumbar lordosis of 47.0°

**Fig. 3:**
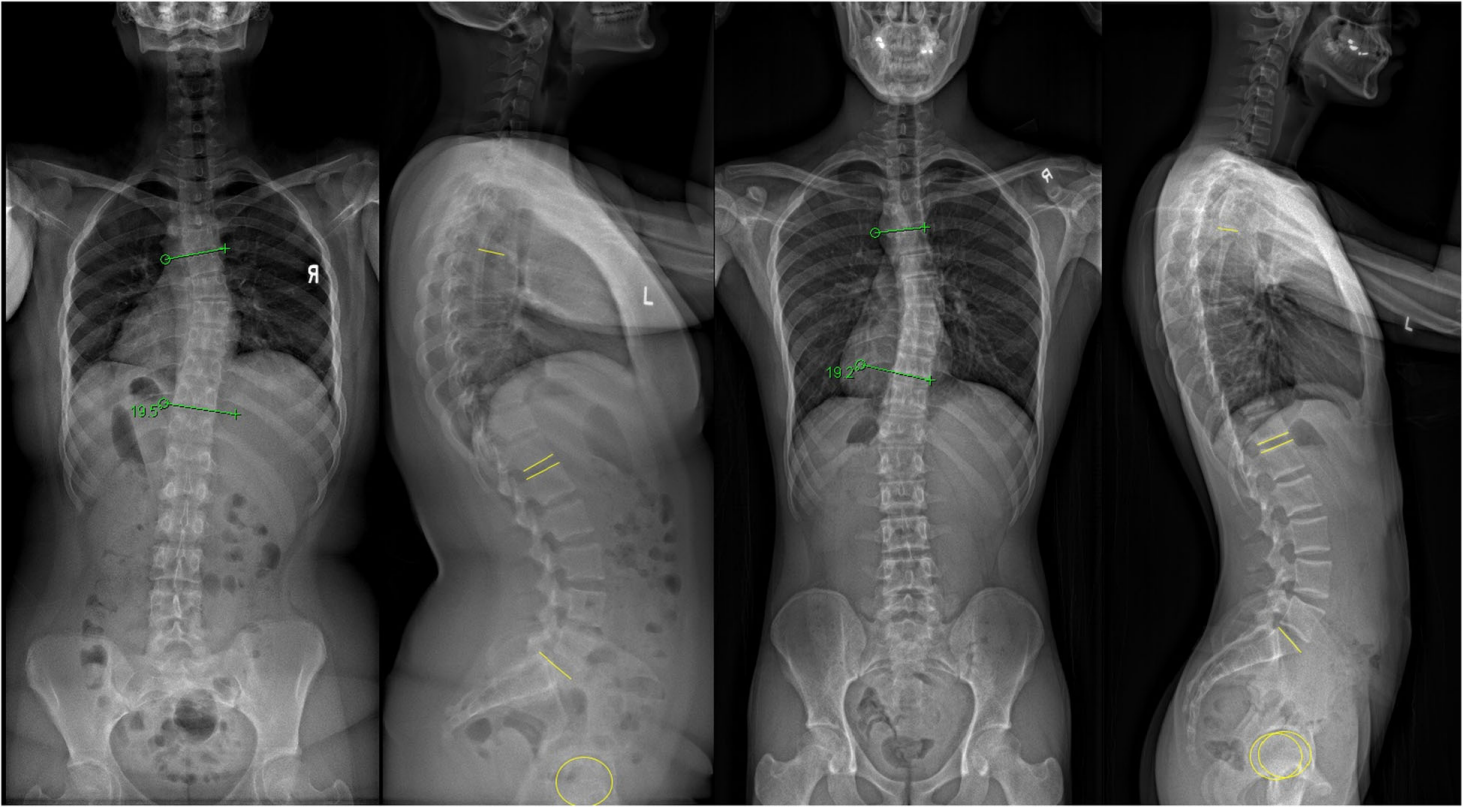
Examples of two patients with similar thoracic coronal deformity but markedly different sagittal alignment. For the first patient with (**a**) thoracic curve of 19.5° at T5-T10, (**b**) sagittal parameters included thoracic kyphosis of 41.9°, lumbar lordosis of 66.2°, pelvic incidence of 42.3°, pelvic tilt of 14.6°, sacral slope of 37.5°, Pelvic incidence – lumbar lordosis of − 14.1°. Despite a similar (**c**) coronal curve magnitude (19.2° at T5-T10), there was a (**d**) greater negative mismatch between pelvic and spinal parameters (− 26.1°) with less thoracic kyphosis (30.2°). Other sagittal parameters included pelvic incidence of 42.9°, pelvic tilt of − 4.1°, sacral slope of 49.3°, and lumbar lordosis of 71.4°

The relationship between coronal curves and sagittal balance in patients with AIS is not well understood. Based on a large study population, we observed large variabilities in coronal and sagittal alignment. The variability in sagittal alignment is apparently independent of coronal curve type and magnitude. Conversely, the PI has a greater influence on sagittal spino-pelvic parameters. Depending on the PI, variations in LL, PT and SS are observed and appear positively correlated. However the TK is consistently hypokyphotic regardless of coronal curve magnitude or degree of PI. Thus coronal and sagittal plane changes should be considered independently and individualized per patient (Figs. 2 and 3). Nevertheless, this study provided the general “norm” in which patients with certain coronal deformity patterns present with in the sagittal plane.

As AIS is a three-dimensional spinal deformity with vertebral rotation, it is common for patients to have a loss in TK due to thoracic structural curves and compensation for sagittal imbalance. The mean value of TK is comparable to other studies [9, 18]. Thoracolumbar/lumbar curves may have more vertebral rotation leading to increases in LL. These coupling relationships are independent from the coronal curve magnitude. Despite different severities of Cobb angle or curve type and location, the TK and LL remains constant. This may suggest that the coronal plane deformity has less influence on the sagittal alignment. It is similar to results from another report [29], albeit smaller sample of 192 subjects, specifically looking at small (< 20°) thoracic curves with or without lumbar curves at an early stage of AIS. The authors observed much less TK in thoracic curves with lumbar curves (27.6° vs 41.9°) as compared to our respective findings (17.3° vs 19.4°). This difference is observed even in our single vs multiple structural curves. Our findings suggest that the sagittal profile variations are less pronounced as the curve size increases. The thoracic hypokyphosis is likely to deteriorate with anterior column growth.

PI is a fundamental component of the “pelvic vertebra” that governs what is acceptable sagittal balance [7]. With increases in LL while the PI remains constant, there is more PI-LL mismatch in thoracolumbar/lumbar curves [30]. Depending on the degree of PI, the entire panel of sagittal spino-pelvic parameters may be altered. The relationship between LL and PI is similar to reports in adults [25]. In the smaller PI group, the LL is comparably much larger. In contrast, the LL matches PI in the large PI group. This is a relationship independent from the major coronal curve deformity. Longitudinal follow-up of these different PI groups is warranted to identify what changes occur with growth. There are growth modulation processes unique to a paediatric population [31] before the PI becomes a static parameter in adults. It is apparent that the sagittal pelvic parameters influence the sagittal alignment more so than the coronal Cobb angle. Yet, the sagittal alignment may be altered by interventions made for coronal curve correction [32]. Hence, monitoring the sagittal alignment should not be neglected.

We expect these features to be a true representation of the curve patterns in AIS. It is unlikely for sagittal decompensation to occur and recruitment of compensatory mechanisms like pelvic retroversion is not observed. Pelvic retroversion is represented by increased PT which maintains the center of gravity over the femoral heads to achieve sagittal balance. The degree of PI is a determination on the possible compensation mechanisms [26]. Patients with larger PI have a larger capacity for pelvic retroversion but requires larger LL [7]. For these patients with AIS, the degree of tolerance appears to be quite high. We expect patients with PI-LL mismatch to have an increase in PT for compensation [33]. With a negative PI-LL mismatch, we expect significant forward bending of the whole sagittal spine to achieve balance. However, despite some patients with low PI presenting with large PI-LL mismatches, there are still no significant changes in pelvic orientation.

In patients with sagittal imbalance, thoracic hypokyphosis is an important compensatory mechanism to maintain balance. This may not explain the patterns observed in the AIS population. These patients are all adolescents who we presume to have normal back musculature. However, since thoracic hypokyphosis occurs in all cases regardless of coronal curve magnitude or PI, we expect this to be a characteristic of the scoliosis deformity rather than compensated sagittal malalignment [9, 18]. Nevertheless, this presentation of cases is important because this group of patients will become adults who may develop adult spinal deformities in the future. Compensatory mechanisms in this background become limited due to the inherent thoracic hypokyphosis and early decompensation may occur as compared to *de novo* degenerative conditions. Nevertheless, the sagittal appearance of these patients with AIS will need reassessment during adulthood.

Despite establishing these relationships on radiographs, changes in quality of life scores appear to rely mostly on the coronal features. It is important to note firstly that although AIS is a common spine problem in adolescents, its effect on the quality of life of patients in general may not be very detrimental. Thus, the SRS-22r questionnaire scores are generally quite high in our study population. Physical aspects, including function and pain, generally have higher scores than the psychological aspects, including appearance and mental health. This shows that AIS causes less confidence in the self-perceived appearance and self-image of patients, even though it has some negative effects on function and pain scores [2, 12]. It seems that different sagittal alignments do not affect the quality of life, while the magnitude of coronal Cobb angle is the main influence of the scores. The greater the coronal Cobb angle, the lower the total score and various domain scores. Importantly, these differences in function and pain domains reached MCID for clinical significance as reported by Carreon et al. [21] The relationship observed between the SRS-22r domain scores and the coronal Cobb angle is compatible with other studies [20, 34]. Older patients seem to have a better self-confidence regarding their appearance, despite greater perceived pain. This may be a result of acceptance of the deformity and development of more chronic muscle imbalance and associated back pain. However, caution is needed when interpreting these minor correlations. The effect of age is likely spurious since there is minimal correlation between age and SRS-22r scores.

There are certain limitations in using two-dimensional radiographic images to examine the condition of patients with AIS. Errors in static images may occur especially with sagittal alignment measurements due to rotational deformities [9]. Hence assessment of the rotational profile is crucial to provide the missing link between coronal and sagittal alignment. The lack of variability with TK is likely a result of axial plane deformity associated with vertebral remodelling of the apical vertebrae [35, 36]. Longitudinal data is necessary to observe the changes that occur with growth [37, 38]. In addition, we have not included the global sagittal parameters which are important for understanding alignment effects on SRS-22r scores. Though, we do not expect global imbalance to be present in AIS as young patients have strong compensatory maneuvers and any hypokyphosis or mismatch between PI and LL should reflect this. We also observed that multiple structural curves had larger Cobb angles than single structural curves. This mainly reflects the problem of a cross-sectional study as it is possible for single structural curves to develop into multiple structural curves with age. Hence, we are unable to verify the importance of multiple curves without longitudinal follow-up. One additional parameter that should be studied in the future is the cervical alignment which as seen from our case examples appear mostly kyphotic.

## Conclusion

Based on a large study population of patients with AIS, we identified several important patterns between coronal and sagittal parameters, and how they indicate the potential compensatory mechanisms. Sagittal spino-pelvic parameters range widely among patients with AIS, and cannot be solely predicted by the coronal deformity. However certain trends with the location of the major curve, curve magnitude and TK have been identified. This along with knowledge of various compensatory mechanisms for sagittal balance in various degrees of PI is elucidated. The TK does not vary with variations in the major curve Cobb angle. The severity of the coronal Cobb angle, reaching a severity of 40°, leads to clinically significant worsened SRS-22r scores.

## Data Availability

The data is kept by the corresponding author and is available upon request.

## Abbreviations

AIS: Adolescent idiopathic scoliosis
ANOVA: Analysis of variance
LL: Lumbar lordosis
MCID: Minimum clinically important difference
PA: Posteroanterior
PI: Pelvic incidence
PI-LL: PI-LL mismatch
PT: Pelvic tilt
SRS-22r: 22-item Scoliosis Research Society questionnaire
SS: Sacral slope
TK: Thoracic kyphosis
